# Biological Effects of Mammalian Translationally Controlled Tumor Protein (TCTP) on Cell Death, Proliferation, and Tumorigenesis

**DOI:** 10.1155/2012/204960

**Published:** 2012-05-22

**Authors:** Michiyo Nagano-Ito, Shinichi Ichikawa

**Affiliations:** Laboratory for Animal Cell Engineering, Niigata University of Pharmacy and Applied Life Sciences (NUPALS), 265-1 Higashijima, Akiha-ku, Niigata-shi, Niigata 956-8603, Japan

## Abstract

Translationally controlled tumor protein (TCTP) is a highly conserved protein found in eukaryotes, across animal and plant kingdoms and even in yeast. Mammalian TCTP is ubiquitously expressed in various tissues and cell types. TCTP is a multifunctional protein which plays important roles in a number of cell physiological events, such as immune responses, cell proliferation, tumorigenicity, and cell death, including apoptosis. Recent identification of TCTP as an antiapoptotic protein has attracted interest of many researchers in the field. The mechanism of antiapoptotic activity, however, has not been solved completely, and TCTP might inhibit other types of cell death. Cell death (including apoptosis) is closely linked to proliferation and tumorigenesis. In this context, we review recent findings regarding the role of TCTP in cell death, proliferation, and tumorigenesis and discuss the mechanisms.

## 1. Introduction

Translationally controlled tumor protein (TCTP) was initially identified as a factor implicated in cell growth [1, 2]. TCTP has also been termed histamine releasing factor (HRF), fortilin, P21, P23, TPT-1, and Q23. This protein was named TCTP because its mRNA was controlled at the translational level [3–5]. Although TCTP is found ubiquitously in tissues and cell types, its expression is relatively low in lung and colon, and cell lines derived from normal cells such as a mouse fibroblast NIH-3T3 and human embryonic kidney HEK293T cells [6]. Because of its multifunctional properties, TCTP has attracted the attention of an increasing number of researchers in many fields (reviewed in [7]). TCTP plays important roles in a number of cell physiological events in cancer, cell proliferation, stress response, gene regulation, and heat shock response [8–13]. TCTP was also shown to possess an extracellular function, that is, histamine release [14].

Tumorigenicity, proliferation, and cell death, including apoptosis, are closely related functions. Uncontrolled or promoted proliferation and loss of cell death are general properties of tumor cells. In this paper, we will focus on mammalian TCTP and discuss its physiological functions, emphasizing cell death, proliferation, and tumorigenesis.

## 2. Properties of TCTP

Human [5] and murine [3] TCTP cDNAs were isolated and their sequences determined more than 20 years ago. Human TCTP cDNA encodes a protein with a calculated molecular mass of 19 kDa (172 amino acids). Sequence analyses revealed that TCTP is a highly conserved protein lacking homology to any other protein. TCTP has been found in a wide range of eukaryotes, including yeast, plants, and animals, suggesting it originated in the distant evolutionary past. Since immune systems are restricted to animals, its function in histamine release has been acquired only recently in evolution. Ubiquitous expression of TCTP in mammalian tissues suggests its importance in normal physiological functions. In fact, a gene-targeting approach revealed that TCTP is an essential protein in mice since knockouts deficient in this protein die at embryonic stage day E9.5-E10.5 [15]. However, studies with mouse embryonic fibroblast (MEF) cells showed that TCTP is not essential for cell survival *in vitro* [15]. The intracellular localization of TCTP is predominantly in the cytosol and nucleus [16] although it functions as an antiapoptotic protein in mitochondria. TCTP is a hydrophilic protein and does not contain any hydrophobic transmembrane domains or any localization signals to an organelle [6]. Translocation of TCTP to the nucleus under certain conditions such as oxidative stress was reported recently. However, TCTP does not contain a nuclear localization signal and the mechanism of translocation remains to be solved [17].

## 3. TCTP Interacts with Many Kinds of Proteins

To exert various physiological functions, TCTP interacts with many other proteins, including translation elongation factors eEF1A and eEF-B-*β* [18], tubulin [19], actin [20], myeloid cell leukemia protein-1 (MCL1) [6, 16], Bcl-xL [21], p53 [22], and Na, K-ATPase [12]. TCTP can also bind to itself, forming homodimers [11], and this binding is required for the cytokine-like activity of this protein during allergic responses [23]. However, it is not known whether the dimerization of TCTP is necessary for its other functions.

## 4. How Does TCTP Protect Cells from Death?

It is well known that TCTP protects cells from death. Although many mechanisms have been proposed, details remain to be identified.

### 4.1. TCTP Could Directly Reduce Cellular Stress

**Figure 1:**
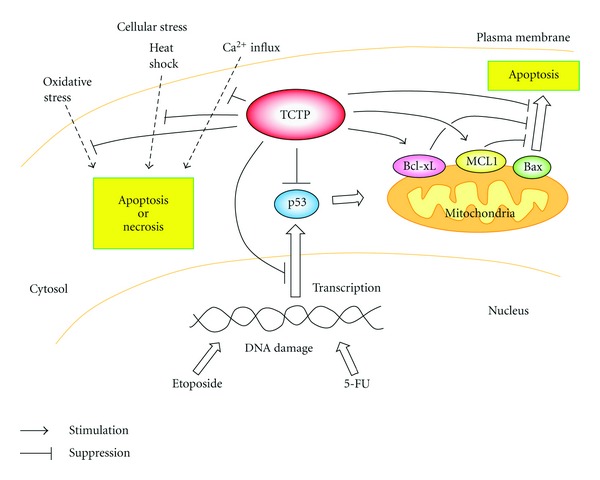
TCTP protects cells from cell death. TCTP inhibits cell death induced by oxidative stress, heat shock, or influx of Ca^2+^. In addition, TCTP can protect cells from apoptosis triggered by treatment with genotoxic reagent such as etoposide and 5-fluorouracil. TCTP inhibits apoptosis by stabilizing antiapoptotic Bcl-2 family proteins, MCL1 and Bcl-xL and by inhibiting activation of proapoptotic Bcl-2 family protein, Bax. Moreover, TCTP inhibits p53-dependent apoptosis by downregulating the protein.

TCTP expression increases in response to a variety of cell stresses and stimuli, and in some cases, TCTP could directly reduce stress, protecting cells from death (Figure 1). The first case we describe deals with its protection of cells from heat shock-induced cell death. TCTP is markedly upregulated in a variety of cells following thermal shock. Recent studies demonstrated that TCTP is a heat shock protein and serves as a molecular chaperone. TCTP binds to denatured proteins, refolds them, and also interacts with native proteins and protects them from denaturation [24]. Although no strong homology with other proteins has been found, recent studies revealed relationships with guanine nucleotide-free chaperones, the Mss4/Dss4 family of proteins that binds to the GDP/GTP-free form of Rab [25]. This fact also supports TCTP's function as a chaperone.

The second case is cell death induced by an influx of Ca^2+^. The level of TCTP is controlled by the intracellular Ca^2+^ concentration and elevation of Ca^2+^ also induces TCTP mRNA in cells [26]. Binding of TCTP to Ca^2+^ was demonstrated for the first time using *Trypanosoma brucei* protein [27] and later with the human protein [28]. Thapsigargin raises cytosolic Ca^2+^ by blocking the ability of the cells to pump calcium into the ER, which depletes its Ca^2+^ stores. This depletion can secondarily activate plasma membrane calcium channels, allowing an influx of Ca^2+^ into the cytosol, thereby initiating apoptosis. The lack of TCTP resulted in exaggerated elevation of Ca^2+^ in thapsigargin-challenged cells [29]. Elevation of the intracellular Ca^2+^ level beyond the normal range could injure mitochondrial membranes and lead to release of cytochrome C and AIF, resulting in apoptosis. Graidist's group also demonstrated that Ca^2+^ binding of TCTP is required for protection of the cells against thapsigargin-induced apoptosis. They hypothesized that TCTP exerts its antiapoptotic function by serving as a Ca^2+^ scavenger. On the other hand, thapsigargin is also known to induce ER stress, in which unfolded proteins are accumulated in the organelle. Thapsigargin reduces Ca^2+^ concentration in the ER and suppresses small molecule Ca^2+^-dependent chaperones in the organelle, allowing accumulation of aberrant proteins, which in turn eventually leads cells to undergo apoptosis. Thus, TCTP might also protect cells from ER stress-induced apoptosis by inhibiting the signal pathway.

The last case is oxidative stress. TCTP from the parasite *Brugia malayi* has antioxidant functions and when it was overexpressed in *Escherichia coli*, it protected the cells from hydrogen peroxide-induced cell death [30]. Although TCTP used in this experiment originated in the parasite and was expressed in bacteria, this result suggests that TCTP itself might serve as antioxidant and could neutralize ROS in mammalian cells.

### 4.2. TCTP Inhibits Apoptosis

Many types of cellular stresses induce apoptosis via the mitochondrial pathway and TCTP is able to inhibit this type of apoptosis by regulating the relevant signal pathways (Figure 1). TCTP protects cells from apoptosis triggered by serum deprivation [6], or treatment with etoposide, taxol, or 5-fluorouracil [21, 31]. Mitochondria contain proapoptotic proteins such as apoptosis inducing factor (AIF), Smac/DIABLO, and cytochrome C. In the course of apoptosis, these proteins are released from mitochondria following the formation of the permeability transition pore in the membrane by the action of proapoptotic Bcl-2 family proteins such as Bax and BH3. Other Bcl-2 family members such as Bcl-2, MCL1, and Bcl-xL are known to suppress apoptosis by binding and inactivating the proapoptotic proteins. Among Bcl-2 family proteins, MCL1 is a unique protein. Unlike other Bcl-2 family proteins, MCL1 is not constitutively expressed and is induced by various stimuli. It was demonstrated that TCTP specifically associates with MCL1 [8, 16], which has the ability to stabilize TCTP [16]. In contrast to this result, another research group showed that TCTP stabilized MCL1 by suppressing its degradation by blocking its ubiquitination [8]. In their experimental conditions, MCL1 did not stabilize TCTP. The discrepancies of the results obtained from the two research groups are presumably due to the use of different cell lines and experimental conditions. TCTP and MCL1 are also capable of functioning as antiapoptotic proteins independently of each other [31]. Bcl-xL is another antiapoptotic Bcl-2 family protein that interacts with TCTP. In this case, binding between the BH3 domain of Bcl-xL and the N-terminal region of TCTP is required for the antiapoptotic activity of TCTP [8]. The BH3 domain is responsible for hetero- and homodimerization between antiapoptotic and proapoptotic Bcl-2 family proteins. TCTP also interferes with dimerization of the proapoptotic Bcl-2 family protein Bax [32]. The crystal structure of TCTP was solved and a structural similarity with that of Bax [32] was found despite lack of amino acid sequence homology. This similarity suggests localization of TCTP to mitochondrial membranes. Dimerization of Bax is required for its apoptotic activity and TCTP blocks the formation of Bax homodimers by inserting into mitochondrial membranes (reviewed in [33]). Although TCTP inhibits apoptosis induced by Bax, unlike MCL1 and Bcl-xL, TCTP does not bind Bax directly.

**Figure 2:**
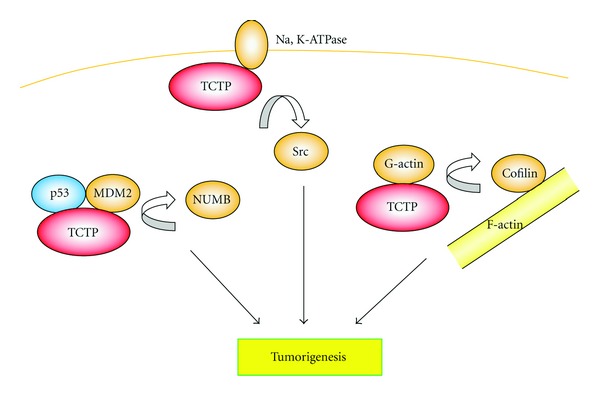
TCTP functions as an oncogene. NUMB forms a tricomplex with p53 and the E3 ubiquitin ligase MDM2, thereby preventing ubiquitination followed by degradation of p53. TCTP promotes p53 degradation by competing with NUMB for MDM2 binding. TCTP binds to Na, K-ATPase and, as a result, it releases Src binding to Na, K-ATPase and activate it. TCTP can release cofilin binding to G-actin by competing with and replacing cofilin. The increase of free cofilin then promotes the binding of the protein to F-actin and exerts its functions.

TCTP affects the tumor suppressor p53 (Figures 1 and 2). The mutation in p53 is found in about half of all cancers and dysfunction of the protein is one of the main causes of cancer development. p53 is also a potent mediator of cellular responses against various cellular stresses including genotoxic insults. In addition, overexpression of p53 induces apoptosis in cancer cells. TCTP was shown to bind p53 and prevent apoptosis by destabilizing the protein in a human lung carcinoma cell line A549 [22]. TCTP also represses transcription of p53 [34]. These facts also indicate the ability of TCTP to promote transformation by reducing p53 function.

### 4.3. Oxidative Stress-Induced Cell Death and TCTP

Intrinsic reactive oxygen species (ROS) such as hydrogen peroxide, superoxide, and hydroxyl radicals are generated in cells in the course of normal metabolism, including electron transport and various oxidase reactions. Oxidative stress induced by ROS has been implicated in aging and in the pathophysiology of various diseases such as diabetes, cancer, and Parkinson's disease (reviewed in [35]). These diseases are, at least in part, caused by ROS-mediated cell death in tissues. Although the effect of TCTP on apoptosis first attracted attention, TCTP might regulate other types of cell death. The types of cell death induced by oxidative stress depend on the cell lines and experimental conditions. In most cases, however, cell death caused by oxidative stress leads to necrosis rather than apoptosis. Types of hydrogen peroxide-induced cell death differ depending on cell types, and conditions of hydrogen peroxide treatment and cell culture. High concentrations of hydrogen peroxide inhibited apoptosis in T-lymphoma Jurkat cells by lowering intracellular ATP levels (necessary for apoptosome formation), and this might also be the case in other cell lines [36]. In the course of isolating cDNAs which protect cells from hydrogen peroxide, we found for the first time that TCTP could inhibit cell death induced by oxidative stress [37]. Overexpression of TCTP protected hydrogen peroxide-induced cell death in a Chinese hamster ovary cell line, CHO-K1; however, cell death was not typical apoptosis. Although the cells showed apoptosis-like morphological changes after hydrogen peroxide treatment, their genomic DNA did not show DNA ladder pattern formation [37]. Presumably the cells stopped apoptotic signaling after cytochrome C release from mitochondria and were subjected to secondary necrosis. Recent studies showed the existence of programmed necrosis (necroptosis) which is physiological cell death regulated by its signal pathway (reviewed in [38]). TCTP might inhibit the signal pathways of physiologically regulated necrosis. As mentioned in the previous section, it is also possible that TCTP itself acts as an antioxidant and reduces oxidative stress induced by hydrogen peroxide. This protective effect of TCTP against oxidative stress is presumably an intrinsic function in malignant breast cancer cells. Treatment with hydrogen peroxide upregulated TCTP level in T4-2 malignant breast cancer cells, but not in their parental S-1 cells that are nonmalignant [39]. TCTP upregulation was also observed in another breast cancer cell line, MDB-MB-231 after treatment with hydrogen peroxide or arsenic trioxide [40], which leads to ROS generation.

 In conclusion, oxidative stress upregulates cellular TCTP levels leading to cellular protection against death. However, hydrogen peroxide treatment did not upregulate TCTP in another tumorigenic cell line (CHO-K1), although over-expression of TCTP protects cells from hydrogen peroxide [37]. The mechanisms by which oxidative stress upregulates TCTP are not known. Interestingly, TCTP translocates from cytosol to the nucleus in a keratinocyte cell line (HaCat) where it binds the vitamin D3 receptor [17]. Thus far, the physiological meaning of TCTP binding to the vitamin D3 receptor is not clear. However, this interesting phenomenon suggests that TCTP might regulate transcription of genes in response to oxidative stress. As the upregulation mediated by hydrogen peroxide is restricted to malignant cancer cells, protein factors controlling its expression could be suitable targets for cancer drug discovery. Primary culture cells of mouse embryonic fibroblasts (MEF) from TCTP knockout and control mice manifested similar proliferative activities and apoptotic sensitivities to various stimuli including hydrogen peroxide treatment [15]. These results suggest that prevention of cell death by TCTP is restricted to certain cell types such as transformed cancer cells. This hypothesis was supported by the fact that the depletion of TCTP by siRNA induced apoptosis via caspases 8 and 3 in human prostate cancer cell line LNCaP [41]. Interestingly, Mmi1P, a yeast ortholog of mammalian TCTP that binds microtubules, translocates from the cytosol to mitochondria following mild oxidative stress stimuli. In contrast to its mammalian counterpart, Mmi P has an apoptotic function in yeast cells [42].

## 5. Tumorigenicity and TCTP

Several lines of evidence indicate that TCTP can induce oncogenic transformation. Transformation of normal cells into tumor cells requires a series of genetic changes. Since TCTP is overexpressed in many types of cancer cells and silencing of the gene decreases the viability of the cells [6], it was postulated that TCTP functions as an oncogene. Tuynder et al. developed unique systems to select cells with a reverted phenotype using H-1 parvovirus which preferentially kills tumor cells [19, 43]. TCTP was found to be downregulated in reverted cells with a normal phenotype. In addition, silencing of TCTP with antisense DNA or siRNA revealed a reverted tumor phenotype, supporting this idea [19, 43, 44]. These results suggest that TCTP is directly involved in malignant transformation. Although the mechanisms of TCTP-dependent transformation are not known, it could be the result of p53 destabilization as noted in the previous section. Another line of evidence also indicates regulation of p53 by TCTP and *vice versa*. NUMB is a protein known to be a regulator of p53. It forms a tricomplex with p53 and the E3 ubiquitin ligase MDM2, thereby preventing ubiquitination followed by degradation of p53 [45]. TCTP promotes p53 degradation by competing with NUMB for MDM2 binding (Figure 2). On the other hand, p53 directly represses transcription of TCTP. Thus, TCTP and p53 form a reciprocal negative regulation loop [34]. This fact also suggests that TCTP might inhibit p53-dependent apoptosis by downregulating the protein.

 The most important properties of tumor and cancer cells are unregulated cell proliferation and avoidance of cell death. Inhibition or gene silencing by TCTP siRNA reduces viability and induces apoptosis in cancer cells, including human prostate cancer cells [41]. TCTP might also be involved in the malignancy of tumors by interacting with actin at the cofilin binding site. Cofilin is an actin binding protein and has the ability to regulate the cell cycle (reviewed in [46]) and promote metastasis [47]. TCTP competes with cofilin at the cofilin-binding site of actin. Although cofilin can bind to both monomeric (G-actin) and filamentous actin (F-actin), it exerts its functions by binding to and changing the twist of F-actin. On the other hand, TCTP has a higher affinity with G-actin than F-actin. TCTP can release cofilin binding to G-actin by competing with and replacing cofilin. The increase of free cofilin then promotes the binding of the protein to F-actin and exerts its functions (Figure 2) [20]. Recent studies also revealed that TCTP induces transformation in human breast epithelial cells through activation of a protooncogene product Src [48]. TCTP binds to the *α*1 subunit of Na, K-ATPase and, as a result, it releases Src binding to Na, K-ATPase. This TCTP-mediated Src release activates Src and promotes various tumor progression signal pathways (Figure 2) [48].

## 6. TCTP Regulates Cell Proliferation

Since TCTP is highly expressed in actively dividing cells [28, 49], one might expect TCTP to modulate physiological functions during cell proliferation. TCTP has the ability to bind microtubules during G1-, S-, G2-, and M-phases of the cell cycle. It associates with the metaphase spindle, but is detached from the spindle after metaphase [50]. TCTP is phosphorylated by the polo-like kinase Plk, which is likely to cause detachment of TCTP from the mitotic spindle [9]. Since the TCTP level is upregulated during entry into the cell cycle, the protein is believed to be important for cell growth and division. TCTP overexpression in mammalian cells results in cell cycle retardation, microtubule stabilization, and alteration of cell morphology [49]. Furthermore, TCTP mutated in the phosphorylation sites for Plk disrupts the completion of mitosis, indicating the importance of TCTP phosphorylation in normal cell cycle regulation [9]. The fact that increased TCTP levels slow cell cycle progression is unexpected because high levels of TCTP expression are generally observed in actively dividing cells and the discrepancy has yet to be explained.

TCTP might regulate proliferation through the target of rapamycin (TOR) pathway. TOR is a Ser/Thr kinase that regulates proliferation and metabolism in response to nutrients, hormones, and growth factors. In case of *Drosophila*, *Dorsophila* TCTP (dTCTP) binds to nucleotide free form of a small GTPase, *Drosophila* Ras homolog enriched in brain (dRheb), and stimulates GDP-GTP exchange of dRheb. As a result, dTCTP activates the TOR signaling pathways. In fact, tissue-specific reduction of dTCTP *in vivo* resulted in smaller organs with reduction of both cell size and cell number [51]. This might be also the case in mammals [52].

## 7. Concluding Remarks

We have reviewed recent findings on biological effects of mammalian TCTP, focusing on inhibition of cell death, regulation of proliferation, and tumorigenesis. Although many hypotheses have been proposed, mechanistic explanations of TCTP on phenomena are still elusive. Presumably, TCTP is able to modulate multiple protein targets simultaneously and as a result, it exerts effects. Further comprehensive studies are necessary to clarify the detailed mechanisms. Recent studies also suggest protective functions of TCTP against cell death other than apoptosis. The mechanisms of TCTP's action on the cell death is interesting and important issues in future studies.
